# *Notes from the Field*: Pan-Resistant New Delhi Metallo-Beta-Lactamase-Producing *Klebsiella pneumoniae* — Washoe County, Nevada, 2016

**DOI:** 10.15585/mmwr.mm6601a7

**Published:** 2017-01-13

**Authors:** Lei Chen, Randall Todd, Julia Kiehlbauch, Maroya Walters, Alexander Kallen

**Affiliations:** ^1^Washoe County Health District, Nevada; ^2^University of Nevada, Reno, ^3^Nevada State Public Health Laboratory, ^4^Division of Healthcare Quality Promotion, National Center for Emerging and Zoonotic Infectious Diseases, CDC.

On August 25, 2016, the Washoe County Health District in Reno, Nevada, was notified of a patient at an acute care hospital with carbapenem-resistant Enterobacteriaceae (CRE) that was resistant to all available antimicrobial drugs. The specific CRE, *Klebsiella pneumoniae*, was isolated from a wound specimen collected on August 19, 2016. After CRE was identified, the patient was placed in a single room under contact precautions. The patient had a history of recent hospitalization outside the United States. Therefore, based on CDC guidance (*1*), the isolate was sent to CDC for testing to determine the mechanism of antimicrobial resistance, which confirmed the presence of New Delhi metallo-beta-lactamase (NDM).

The patient was a female Washoe County resident in her 70s who arrived in the United States in early August 2016 after an extended visit to India. She was admitted to the acute care hospital on August 18 with a primary diagnosis of systemic inflammatory response syndrome, likely resulting from an infected right hip seroma. The patient developed septic shock and died in early September. During the 2 years preceding this U.S. hospitalization, the patient had multiple hospitalizations in India related to a right femur fracture and subsequent osteomyelitis of the right femur and hip; the most recent hospitalization in India had been in June 2016.

Antimicrobial susceptibility testing in the United States indicated that the isolate was resistant to 26 antibiotics, including all aminoglycosides and polymyxins tested, and intermediately resistant to tigecycline (a tetracycline derivative developed in response to emerging antibiotic resistance). Because of a high minimum inhibitory concentration (MIC) to colistin, the isolate was tested at CDC for the *mcr-1* gene, which confers plasma-mediated resistance to colistin; the results were negative. The isolate had a relatively low fosfomycin MIC of 16 *μ*g/mL by ETEST.* However, fosfomycin is approved in the United States only as an oral treatment of uncomplicated cystitis; an intravenous formulation is available in other countries.

A point prevalence survey, using rectal swab specimens and conducted among patients currently admitted to the same unit as the patient, did not identify additional CRE. Active surveillance for multidrug-resistant bacilli including CRE has been conducted in Washoe County since 2010 and is ongoing; no additional NDM CRE have been identified.

This report highlights three important issues in the control of CRE. First, although CRE are commonly sent to CDC as part of surveillance programs or for reference testing, isolates that are resistant to all antimicrobials are very uncommon. Among >250 CRE isolate reports collected as part of the Emerging Infections Program, approximately 80% remained susceptible to at least one aminoglycoside and nearly 90% were susceptible to tigecycline (*2*). Second, to slow the spread of bacteria with resistance mechanisms of greatest concern (e.g., gene encoding NDM or *mcr-1*) or with pan-resistance to all drug classes, CDC recommends that when these bacteria are identified, facilities ensure that appropriate infection control contact precautions are instituted to prevent transmission and that health care contacts are evaluated for evidence of transmission (*3*). Third, the patient in this report had inpatient health care exposure in India before receiving care in the United States. Health care facilities should obtain a history of health care exposures outside their region upon admission and consider screening for CRE when patients report recent exposure outside the United States or in regions of the United States known to have a higher incidence of CRE (*1*).

## Footnotes

* http://www.biomerieux-diagnostics.com/etest.
